# Overexpression of Histone Deacetylase 6 Enhances Resistance to Porcine Reproductive and Respiratory Syndrome Virus in Pigs

**DOI:** 10.1371/journal.pone.0169317

**Published:** 2017-01-04

**Authors:** Tianyu Lu, Zhiyuan Song, Qiuyan Li, Zhiguo Li, Meng Wang, Lin Liu, Kegong Tian, Ning Li

**Affiliations:** 1 State Key Laboratories of Agro-biotechnology, College of Biological Science, China Agricultural University, Beijing, China; 2 State Key Laboratory of Medicinal Chemical Biology, Collaborative Innovation Center for Biotherapy, Department of Genetics and Cell Biology, College of Life Sciences, Nankai University, Tianjin, China; 3 National Research Center for Veterinary Medicine, High-Tech. District, Luoyang, Henan Province, China; 4 College of Animal Science and Veterinary Medicine, Henan Agricultural University, Zhengzhou, Henan Province, China; Wageningen Universiteit en Researchcentrum IMARES, NETHERLANDS

## Abstract

Porcine reproductive and respiratory syndrome virus (PRRSV) is one of the most economically relevant viral pathogens in pigs and causes substantial losses in the pig industry worldwide each year. At present, PRRSV vaccines do not effectively prevent and control this disease. Consequently, it is necessary to develop new antiviral strategies to compensate for the inefficacy of the available vaccines. Histone deacetylase 6 (HDAC6) is an important member of the histone deacetylase family that is responsible for regulating many important biological processes. Studies have shown that HDAC6 has anti-viral activities during the viral life cycle. However, whether *HDAC6* overexpression enhances resistance to PRRSV in pigs remains unknown. In this study, we used a somatic cell cloning method to produce transgenic (TG) pigs that constitutively overexpress porcine *HDAC6*. These TG pigs showed germ line transmission with continued overexpression of HDAC6. *In vitro*, virus-challenged porcine alveolar macrophages (PAMs) overexpressed *HDAC6*, which suppressed viral gene expression and PRRSV production. *In vivo*, resistance to PRRSV in TG pigs was evaluated by direct or cohabitation mediated infection with a highly pathogenic PRRSV (HP-PRRSV) strain. Compared with non-TG (NTG) siblings, TG pigs showed a significantly lower viral load in the lungs and an extended survival time after infection with HP-PRRSV via intramuscular injection. In the cohabitation study, NTG pigs housed with challenged NTG pigs exhibited significantly worse clinical symptoms than the other three in-contact groups. These results collectively suggest that HDAC6 overexpression enhances resistance to PRRSV infection both *in vitro* and *in vivo*. Our findings suggest the potential involvement of HDAC6 in the response to PRRSV, which will facilitate the development of novel therapies for PRRSV.

## Introduction

Porcine reproductive and respiratory syndrome (PRRS) is one of the most severe infectious diseases that threaten the swine industry worldwide. PRRS has caused substantial economic losses in swine production [1]. The causative agent of this syndrome is PRRS virus (PRRSV), which is classified in the family *Arteriviridae* within the order *Nidovirales*. PRRSV has a positive-sense, single-stranded RNA genome that is approximately 15.4 Kb in length [2]. PRRS is characterized by abortion, premature birth, and fetal death in pregnant sows and by severe respiratory failure in nursery pigs [3]. In 2006, the highly pathogenic PRRSV (HP-PRRSV) emerged in China, with a unique molecular hallmark of a discontinuous deletion of 30 amino acids in nonstructural protein 2 (Nsp2). HP-PRRSV infection is characterized by high fever, high morbidity, and high mortality in pigs of all ages [4]. The emergence of HP-PRRSV exacerbates the international risks associated with PRRS.

At present, vaccination is the most common method used to control and prevent PRRSV infection. However, because of the high genetic variability of the PRRSV genome and because of the complexity of its infection mechanism, there are many drawbacks to currently available commercial vaccines [5–7]. Thus, there is an urgent demand for novel strategies to control PRRSV infection and transmission.

Histone deacetylase (HDAC) family member proteins show obvious subcellular localization, with most enzymes primarily localizing to the cell nucleus [8]. However, as it contains a cytoplasmic retention signal, histone deacetylase 6 (HDAC6) localizes exclusively to the cytoplasm [9,10]. Unlike other members of the HDAC family, HDAC6 has histone deacetylase activity only *in vitro* [10]. *In vivo*, HDAC6 deacetylates non-histone proteins, such as α-tubulin, heat shock protein 90 (Hsp90) and cortactin [11–13]. Via its deacetylase activities with these substrates, HDAC6 plays an important role in a variety of biological processes, including cell morphology, cell migration, immune synapse formation, and misfolded protein degradation [14–18]. Studies have reported that HDAC6 is involved in a variety of viral life cycles, including human immunodeficiency virus type 1 (HIV-1) [19], human T-lymphotropic virus type-1 (HTLV-1) [20], vesicular stomatitis virus (VSV) [21], and influenza A virus (IAV) [22–24]. Moreover, most of these studies have shown that HDAC6 overexpression enhances resistance to viral infection in cells and in TG mice. However, whether HDAC6 is involved in the PRRSV life cycle remains to be determined.

In this study, we generated *HDAC6*-overexpressing pigs using somatic cell nuclear transplantation (SCNT). We observed that *HDAC6* overexpression inhibited PRRSV infection *in vitro* and enhanced resistance to PRRSV *in vivo*. Furthermore, we observed that acetylated α-tubulin (Actub) levels were elevated in PRRSV-infected MARC-145 and PAMs and that *HDAC6* overexpression suppressed the elevation of Actub in PRRSV-infected cells. These results suggest the potential involvement of HDAC6 and the cytoskeleton in response to PRRSV and will facilitate the development of innovative PRRSV therapies.

## Materials and Methods

### Ethics statement

This study was carried out in strict accordance with the recommendations in the Guide for the Care and Use of Laboratory Animals in China. The protocol was approved by the Committee on the Ethics of Animal Experiments of China Agricultural University (Permit Number: SKLAB-2012-04-07). Forty-two F1 pigs (Landrace, aged from six to eight weeks old, weighing 7–10 kg; more details in S1 Table) were used in present study. The animals were acclimated before use and were housed in filtered ventilated cages. The pigs were provided HEPA-filtered air, pig diet and tap water ad libitum. The environmental conditions included a controlled light cycle (9 h light), temperature (20–26°C) and air humidity (40%-60%). The general condition of pigs was directly monitored by veterinary technicians/trained animal care staff every hour. Any animal that displayed behaviors indicating excessive infection was immediately euthanized via CO_2_ asphyxiation. PRRSV-uninfected control pigs (n = 10) were euthanized by lethal CO_2_ overdoses at 19 days post infection (dpi). The pigs used for obtaining PAMs were administered a sodium pentobarbital overdose of 20 mg per kilogram of weight.

### Cells and viruses

Marc-145 cells, a PRRSV-permissive cell line, were maintained in Dulbecco’s modified Eagle’s medium (DMEM) (Gibco, Cat. 11995–073) supplemented with 10% fetal bovine serum (FBS) at 37°C with 5% CO_2_. PAMs were obtained using lung lavage, as previously described [25]. The PAMs were then grown in RPMI-1640 supplemented with 10% FBS and penicillin-streptomycin (Gibco, Cat. 15140–122) at 37°C with 5% CO_2_. Marc-145 cells were transfected with DNA constructs using an Amaxa Nucleofector Kit (Lonza) according to the manufacturer’s instructions.

Two PRRSV strains were used in the present study to infect cells and pigs: CH-1a (GenBank accession No. AY032626) and JXA1 (GenBank accession No. EF1122445). CH-1a (a gift from Prof. Wenhai Feng of China Agriculture University) was the first PRRSV strain isolated in China. JXA1 is a highly pathogenic porcine reproductive respiratory syndrome (HP-PRRSV) strain that was isolated in Jiang Xi Province, China in 2006 and that has homology with JXwn06 [4]. The PRRSV strains were grown and titrated as previously described [26].

### Generation of transgenic pigs

**Fig 1:**
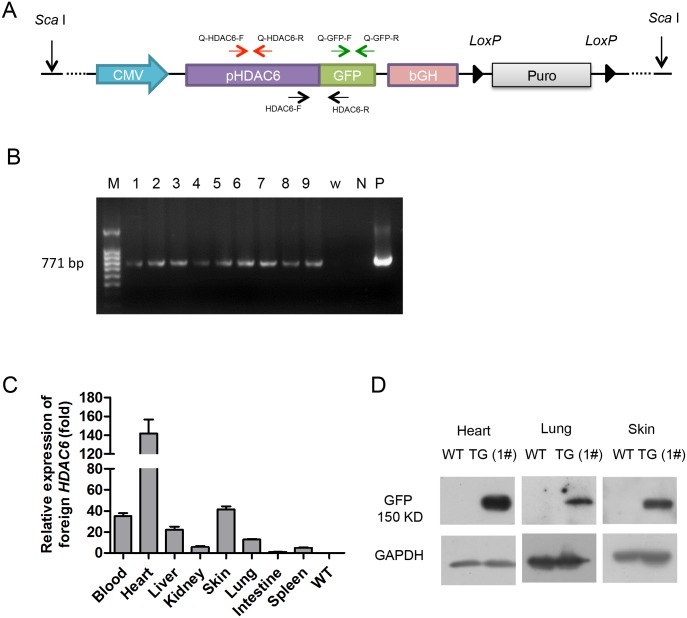
Production and identification of F0 TG pigs. (A) Schematic diagram of the transgenic vector (pCMV-pHDAC6-Puro). The pair of black arrows indicates the primers used to identify the transgenic insert in TG pigs and in fibroblast colonies. The pair of green arrows indicates the primers used to identify exogenous *HDAC6* mRNA in pigs. The pair of red arrows indicates the primers used to determine total *HDAC6* mRNA levels in F1 pigs. (B) PCR assay to detect founder transgenic pigs. The PCR product (771 bp) was the GFP tag of the inserted transgene, which was amplified using the HDAC6-F/R primer pair. M, 100 bp DNA ladder; Lanes 1–9, founders No. 1–9; w, water; P, plasmid control; N, wild-type pig genomic DNA, used as the negative control. (C) qRT-PCR analysis of exogenous *HDAC6* expression in different tissues (blood, liver, kidney, skin, lung, intestine and spleen) of the F0 transgenic pig using the Q-GFP-F/R primer pair. The blood sample data are presented as the mean±SD from 3 individuals (No. 1, 2 and 3). The data from other tissues are presented as the mean±SD from 3 repeated experiments. RNA from the intestines was used as the reference sample. WT, wild-type control. (D) Western blot analysis of F0 transgenic pigs. The samples were collected from tissues of TG (No.1) and WT pigs. The protein samples were probed with an anti-GFP antibody.

The vector overexpressing pig *HDAC6* was termed pCMV-pHDAC6-Puro (Fig 1A). In this vector, the cDNA of the pig *HDAC6* gene is fused to the green fluorescent protein (*GFP*) coding sequence, and cDNA expression is driven by the cytomegalovirus (CMV) promoter. The eukaryotic selection marker in this vector is puromycin, which is flanked by *LoxP* sites. Porcine fetal fibroblasts derived from Landrace pigs were established and cultured as previously described [27]. Fibroblasts were transfected with linearized pig *HDAC6* expression vectors (digested with *Sca*I) using an Amaxa Nucleofector Kit (Lonza) according to the manufacturer’s instructions. After 24 h, the cells were transferred to six 10-cm dishes containing selective medium supplemented with puromycin (Sigma, Cat. P8833) (1 μg/mL). After 5 days, the puromycin-resistant colonies were selected and expanded, and a small sample from each clone was analyzed by polymerase chain reaction (PCR) using the following primers: HDAC6-F, 5’-TCTTCTTCAAGGACGACGGCAACT-3’; HDAC6-R, 5’-GCATGCCTGCTATTGTCTTCCCAA-3’. Positive clones were used as donors for somatic cell nuclear transplantation (SCNT). The cloned pigs were born via natural birth after approximately 106 days, and these TG founder pigs (F0 generation) were artificially inseminated with wild-type (WT) semen to produce the F1 generation. The genotypes of the TG founder pigs and their offspring were verified via PCR analysis of genomic DNA derived from ear biopsies using the HDAC6-F and HDAC6-R primers.

### RNA isolation and qRT-PCR

Total RNA was extracted from tissues and cells using an RNeasy Fibrous Tissue Mini Kit (Qiagen, Cat. 74704) according to the manufacturer’s instructions. MMLV reverse transcriptase (Promega, Cat. 28025–013) was used for reverse transcription according to the manufacturer’s instructions, and qRT-PCR analysis was performed using the Roche Light Cycler 480 System (LC 480; Roche, Basel, Switzerland). The amplifications were performed in 15-μL reactions containing 1 μL template DNA, 0.15 μL each primer (10 μM/μL), 7.5 μL Power SYBR Green Mix (Roche, Cat.4887352001), and 6.2 μL ddH_2_O. All reactions were performed using the following cycle conditions: one cycle at 95°C for 10 min; 40 cycles at 95°C for 10 s, 60°C for 10 s and 72°C for 10 s; one cycle at 95°C for 5 s and 65°C for 1 min. A final cycle at 97°C was performed to generate a melting curve. Two primer pairs were used to quantify *HDAC6* expression. One pair binds to the *HDAC6* sequence and was used to detect the level of total *HDAC6* mRNA in pigs, while the other primer pair binds to the GFP sequence and was used to detect the level of exogenous *HDAC6* mRNA. The relative expression of HADC6 was analyzed as described in previous studies [28–30]. The relative expression of *GFP* was calculated using the 2^-Δct^ method. The relative mRNA expression of total *HDAC6* was calculated using the 2^-ΔΔct^ method, RNA from NTG pigs was used as the reference sample. Glyceraldehyde-3-phosphate dehydrogenase (*GAPDH*) was used as an internal control for qRT-PCR. The following real-time PCR primer pairs were used: Q-HDAC6-F, 5’-CTCGGGAGGTGACAACCAGA-3’ and Q-HDAC6-R, 5’-CTTT TGGGGACTCCTGAACC-3’; Q-GFP-F, 5’-CGACCACTACCAGCAGAACA-3’ and Q-GFP-R, 5’-TCCAGCAGGACCATGTGATC-3’; Q-GAPDH-F, 5’-ATCAC CATCTTCCAGGAGCGA-3’ and Q-GAPDH-R, 5’-AGCCTTCTCCATGGTCGTGAA-3’.

### Western blot analysis

The cells and tissues were lysed in immunoprecipitation (IP) lysis buffer (Beyotime Institute of Biotechnology, China). The protein concentrations of the samples were measured using bicinchoninic acid (BCA) assays (Beyotime). Equal amounts of each of the samples were separated using sodium dodecyl-sulphate polyacrylamide gel electrophoresis (SDS-PAGE) and then transferred onto polyvinylidene difluoride (PVDF) membranes.

The membranes were probed using mouse anti-acetylated α-tubulin (1:10,000 dilution) (Sigma-Aldrich, Cat. T7451), anti-α-tubulin (1:1000 dilution, Santa Cruz, Cat. sc-23948), anti-GFP (1:1000 dilution, Abmart, Cat. M20004) or rabbit anti-GAPDH (1:5000 dilution, Cell Signaling, Cat. 2118) primary antibodies. HRP-conjugated goat anti-mouse (1:20,000 dilution, ZSGB-Bio, Cat. ZDR-5307) or goat anti-rabbit (1:20,000 dilution, ZSGB-Bio, Cat. ZDR-5306) secondary antibodies were then used for signal detection.

The PVDF membranes were then processed using a Super Signal West Pico Chemiluminescent Kit (Thermo Scientific, Cat. 34080) and exposed to autoradiography film. The protein bands were quantified using Gel-Pro Analyzer software (Media Cybernetics, USA). Increases in α-tubulin acetylation were quantified and expressed as the ratio of the intensity of the acetylated α-tubulin band to the intensity of the GAPDH band (Ac/GAPDH).

### *In vitro* infection of PAMs with PRRSV

PAMs were obtained using post-mortem lung lavages in two pigs from the F1 generation that had the same parents but different genotypes.

Viral infections were performed using PRRSV CH-1a or JXA1 strains at MOIs of 0.05 or 0.25, respectively. The PAMs and culture medium were harvested at 24, 48, and 72 hpi.

qRT-PCR was performed to detect PRRSV RNA using primers designed against the conserved region of *ORF7* in PRRSV, as previously reported [31,32,26]. The following primers were used for qRT-PCR: ORF7-F, 5’-AATAACAACGGCAAGCAGCA-3’; ORF7-R, 5’-GCACAGTATGATGCGTCGGC-3’. *GAPDH* served as an internal control gene. The ΔΔCt method for relative quantitation of gene expression was used to determine viral RNA levels. The ΔCt was calculated by subtracting the Ct for GAPDH from the Ct for viral ORF7 RNA. The ΔΔCt was calculated by subtracting the ΔCt for the reference sample (RNA of NTG PAMs at 24 hpi) from the ΔCt for each sample.

In addition, absolute qRT-PCR was conducted to detect the copy number of PRRSV in the supernatant. Viral RNA in the supernatant was then isolated from the culture media using a QIAamp Kit (Qiagen, Cat. 52906). A standard set of mixtures (representing 10^9^, 10^8^, 10^7^, 10^6^, 10^5^, and 10^4^ copies of plasmid DNA consisting of the *ORF7* sequence as previously described [32]) was used to generate a standard curve to determine the correlation between the Ct value and the virion copy number. The primers used for detecting the PRRSV copy number were CH-1a-F (5’-TTCCTCTAGCGACCGAAGATGAC-3’) and CH-1a-R (5’-TGGATCGACGACAGACACAATTG-3’).

### *In vivo* infection and transmission studies in pigs

Before virus challenge, all pigs from the F1 generation were confirmed to be negative for PRRSV infection. The pigs were divided into three separate rooms. Six *HDAC6* TG pigs and six NTG sibling pigs were directly infected with 3 mL (3×10^2.5^ TCID_50_) of the HP-PRRSV strain JXA1 by intramuscular injection. These pigs were the challenged groups. The TG challenged group was housed in room 1, and the NTG challenged group was housed in room 2. Each challenged group was housed with five TG pigs and five NTG pigs per room (i.e., room 1 and room 2 consisted of 16 pigs housed together in an isolated room from dpi = 0). The pigs that were housed with the challenged pigs were raised together in the same cages to ensure physical contact. These housed pigs were not injected in order to mimic the natural spread of the virus through cohabitation. The in-contact experimental pigs consisted of 4 in-contact groups: TG pigs housed with challenged TG pigs (TG/TG), NTG pigs housed with challenged TG pigs (NTG/TG), TG pigs housed with challenged NTG pigs (TG/NTG), and NTG pigs housed with challenged NTG pigs (NTG/NTG). In addition, five TG pigs and five NTG pigs were injected with PBS using the same dose volume and route used for viral infections; these pigs served as the unexposed controls. As shown in S2 Table, all animals were serologically tested and found to be negative for the PRRSV antibody prior to infection.

Rectal temperatures and body weights were monitored daily until death. Blood samples were collected at 0, 2, 4, 6, 8, 11, 14, 17 and 19 dpi. Serum samples were tested using commercial PRRSV enzyme-linked immunosorbent assays (ELISAs; IDEXX Laboratories, Inc., Westbrook, ME, USA) to detect antibodies against PRRSV.

All animal studies were performed in accordance with protocols approved by the animal welfare committee of China Agricultural University, and all efforts were made to minimize potential pain and distress. The humane endpoint was used for all pigs displaying the clinical symptoms of PRRS. The pigs were euthanized under anesthesia if severe disease symptoms were observed, such as being unable to eat or drink, or if the animals were deemed moribund. The animals that were euthanized on a given day were recorded as deaths for that day.

### Quantification of viral RNA load in the serum and lung tissues of pigs

The following assays were conducted as previously described [25]. Briefly, RNA was extracted using a QIAcube System (Qiagen) to quantify the viral RNA load in isolated serum (100 μL) and lung tissues (1 mg). The RT and Q-PCR reactions were performed using a PRRSV Q-PCR Kit (Beijing Anheal Laboratories Co., Ltd.) according to the manufacturer’s instructions.

To detect the viral load in each sample, a standard curve was generated using 10-fold serial dilutions of the viral lysates. These lysates ranged from 10^6^ to 10^2^ TCID_50_ and were used to determine the correlation between the Ct value and TCID_50_.

### Statistical analysis

The results were analyzed using one-way ANOVA and Student’s t-test using GraphPad Prism software (GraphPad Software, San Diego, CA, USA). A P-value of less than 0.05 was considered statistically significant.

## Results

### Generation and identification of transgenic pigs

As shown in Fig 1A, we generated a vector that expressed porcine *HDAC6* fused to *GFP*. Using this vector, we produced *HDAC6* overexpressing TG pigs via SCNT. Nine TG-positive female founders (F0) were obtained from three surrogates (Fig 1B). To verify that the TG pigs had increased transcription of *HDAC6*, quantitative real-time PCR (qRT-PCR) was conducted. One founder pig (No. 1 in Fig 1B) was sacrificed to examine exogenous *HDAC6* expression in various organs (liver, kidney, skin, lung, intestine and spleen), and three F0 pigs (No. 1, 2, and 3) were used to examine exogenous *HDAC6* expression in blood samples. As expected, exogenous *HDAC6* mRNA was detected in the TG pigs, and exogenous *HDAC6* mRNA expression levels varied across the tissues examined in the TG pig (Fig 1C). Because the TG protein is a fusion protein with a GFP tag, an assessment of HDAC6 overexpression was examined using an anti-GFP antibody. Similar to the qRT-PCR results, GFP protein expression was detected only in heart, lung and skin tissues of the TG pigs (Fig 1D).

**Fig 2:**
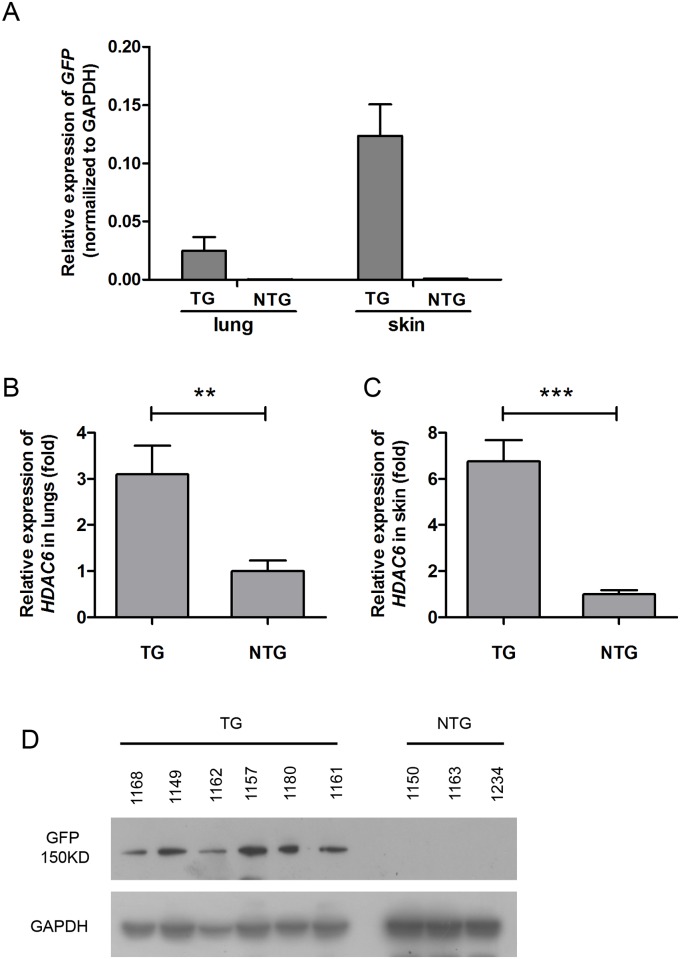
Determination of HDAC6 expression in F1 generation transgenic pigs. (A) qRT-PCR analysis of exogenous *HDAC6* expression in the lungs and skin of F1 TG (n = 9) and sibling NTG pigs (lung, n = 10; skin, n = 6) using the Q-GFP-F/R primer pair. The data are presented as the mean ± SD. The relative expression of *GFP* was calculated using the 2^-Δct^ method. (B) qRT-PCR analysis of *HDAC6* expression in lungs of F1 TG (n = 9) and sibling NTG pigs (n = 10) using the Q-HDAC6-F/R primer pair. (C) qRT-PCR analysis of HDAC6 expression in the skin of F1 TG (n = 9) and sibling NTG pigs (n = 6) using the Q-HDAC6-F/R primer pair. The data are presented as the mean±SD. The mRNA relative expression of total HDAC6 was calculated using the 2^-ΔΔct^ method (RNA from NTG pigs was used as the reference sample). GAPDH was used as an internal qRT-PCR control. Statistical significance was analyzed using a t-test. ***, P<0.001 **; P<0.01. (D) Western blot analysis of F1 pigs. The samples were collected from lung biopsies of six TG pigs and three sibling NTG pigs. The protein samples were probed with an anti-GFP antibody. GAPDH was used as an internal control for western blot analysis.

When the pigs were sexually mature, positive founders were artificially inseminated with WT sperm to produce 80 piglets (the F1 generation). Of these piglets, 43 were *HDAC6*-TG and 37 were NTG progeny. In lung and skin tissue samples obtained from F1 generation pigs, exogenous *HDAC6* mRNA was detected using the Q-GFP-F/R primer pair (Fig 2A). Additionally, *HDAC6* expression was assessed in lung and skin tissues obtained from F1 generation pigs using the primer pair Q-HDAC6-F/R. The results showed that the level of *HDAC6* mRNA in the lung and skin tissues of the TG pigs was approximately two- or six-fold higher than in the NTG siblings, respectively (Fig 2B and 2C). As expected, GFP expression was detected in the lungs of TG pigs but not in their NTG siblings (Fig 2D and S1 Fig).

Moreover, western blot analysis was performed to detect the levels of acetylated α-tubulin (Actub), which is a substrate of HDAC6, in order to examine HDAC6 deacetylase activity. Reduced levels of Actub in the lungs of TG pigs suggested that HDAC6 enzymatic activity was upregulated (S2A and S2B Fig). Taken together, these results indicate that the TG pigs had higher deacetylase enzyme activity than the WT pigs and that this effect was transmitted to the F1 generation. Both genotypes of progeny from the F1 generation were used in the challenge studies described below.

### PRRSV replication inhibited in PAMs isolated from TG pigs

**Fig 3:**
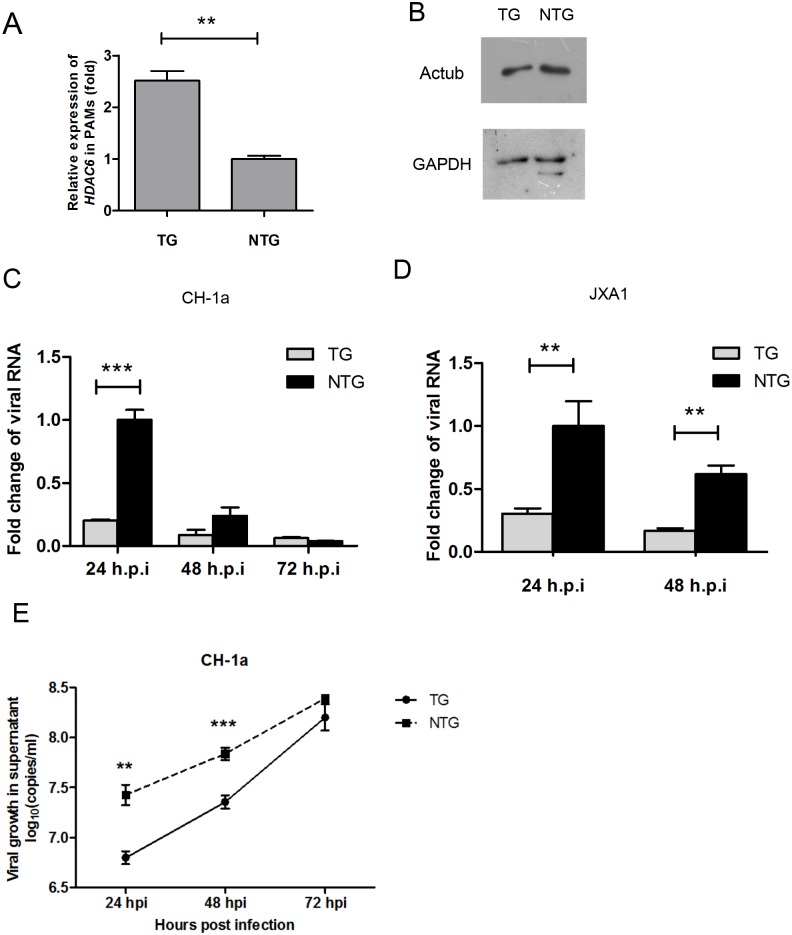
*HDAC6* Overexpression inhibits viral gene expression and PRRSV production in PAMs. (A) qRT-PCR analysis of *HDAC6* expression in PAMs isolated from pigs using the Q-HDAC6-F/R primer pair. *HDAC6* expression is presented as a ratio relative to the level of *GAPDH*. The data are presented as the mean±SD from three independent experiments. (B) Western blot analysis of PAMs isolated from F1 pigs. The protein samples were probed with anti-Actub and GAPDH antibodies. (C) qRT-PCR analysis of viral *ORF7* RNA levels in TG and NTG PAMs that were inoculated with the PRRSV strain CH-1a (MOI = 0.5) for 24, 48 and 72 h. The data represent the results of three independent experiments (mean±SD). (D) qRT-PCR analysis of viral *ORF7* RNA levels in TG and NTG PAMs that were inoculated with the PRRSV strain JXA1 (MOI = 0.25) for 24 and 48 h. The data are presented relative to the expression of *GAPDH* mRNA and represent the results of three independent experiments (mean±SD). RNA from NTG PAMs at 24 hpi was used as the reference sample. Statistical significance was analyzed using a t-test. *, P<0.05; **, P<0.01; ***; P<0.001. (E) The supernatant containing PRRSV RNA was analyzed based on absolute quantitative RT-PCR values at the indicated time points. PAMs were infected with PRRSV CH-1a (MOI = 0.05), and the supernatant was collected and used for RNA extraction and absolute qPCR analysis of the virions at 24, 48 and 72 hpi. The data are representative of the results of three independent experiments (mean±SD). Statistical significance was analyzed using Student’s t-test. **, P<0.01; ***, P<0.001.

To explore whether *HDAC6* overexpression influences PRRSV replication, we analyzed PRRSV replication in PAMs, which are the target cells of PRRSV. We first examined *HDAC6* mRNA expression levels in PAMs. As shown in Fig 3A, the *HDAC6* mRNA expression levels in TG PAMs were approximately two times higher than in NTG PAMs. Consistent with these results, the reduced levels of Actub observed in TG PAMs also suggest that HDAC6 is overexpressed in TG PAMs (Fig 3B).

Next, PAMs were infected with either the CH-1a or JXA1 strain of PRRSV. Intracellular viral replication was analyzed at 24, 48, and 72 h post-infection (hpi). Viral growth in TG PAMs infected with CH-1a was approximately 4-fold lower at 24 hpi and 2.7-fold lower at 48 hpi than the level observed at corresponding time points in NTG PAMs. At 72 hpi, the two groups showed similar levels of viral RNA, and viral RNA was barely detected (Fig 3C). Similar results were obtained in PAMs infected with the JXA1 strain (Fig 3D), where the level of viral RNA in NTG PAMs was approximately 3 times higher than in TG PAMs at 24 and 48 hpi. We did not harvest cells at 72 hpi because significant cell lysis had occurred by that time point. Viral RNA was isolated from the apical media collected from CH-1a-infected cells. ORF7 levels in supernatants were analyzed by qRT-PCR. Consistent with intracellular virus replication, NTG PAMs released more virions into the supernatant than TG PAMs (Fig 3E). These results indicate that PRRSV replication was inhibited in TG PAMs.

### Transgenic pigs exhibit resistance to HP-PRRSV

**Fig 4:**
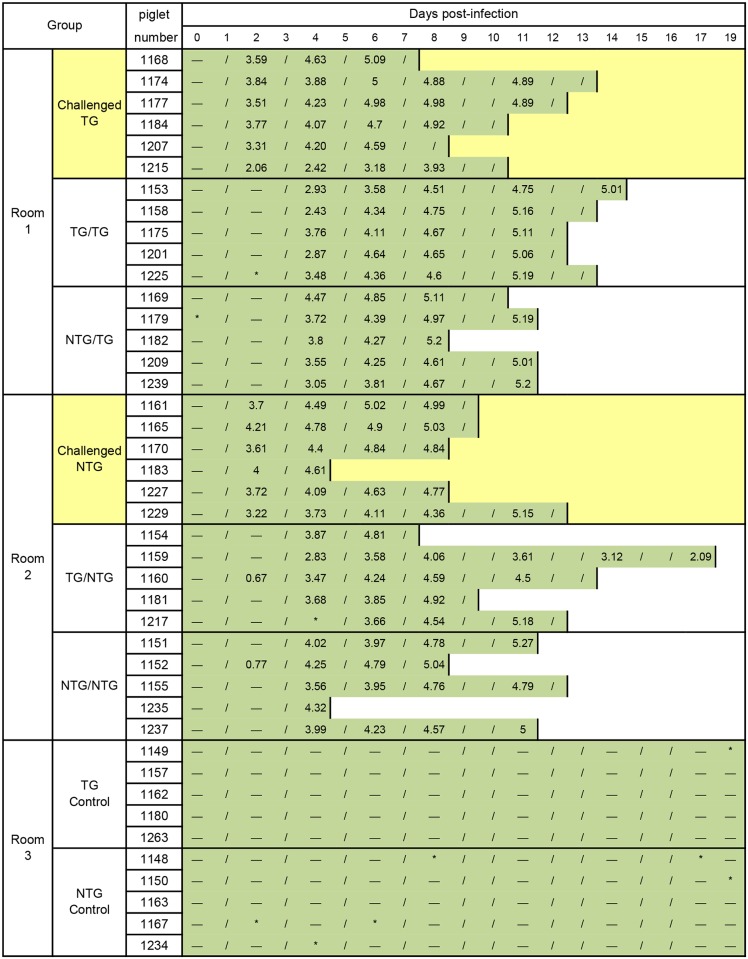
Mortality and viral load data for the challenge and in-contact groups in the study. Two groups of six TG pigs or six NTG pigs were “challenged” groups that were infected with JXA1 on day 0 and then housed with five TG and five NTG “in-contact” pigs, respectively. The length of the green bar indicates survival times. The numbers for each day represent the estimated viral titer in blood samples, which is expressed as log10 TCID_50_/100 μL. “-” indicates that no virus was detected, “*” indicates that blood sample was damaged, and “/” indicates that the blood sample was not collected.

Resistance to PRRSV in TG pigs was evaluated by experiments in which pigs were infected with the HP-PRRSV strain JXA1. TG and NTG sibling pigs were randomly distributed and housed in three separate rooms (Fig 4, the design of the animal experiment described in the Materials and Methods). As shown in S1 Table, all animals were serologically tested and found to be negative for PRRSV antibody prior to infection.

**Fig 5:**
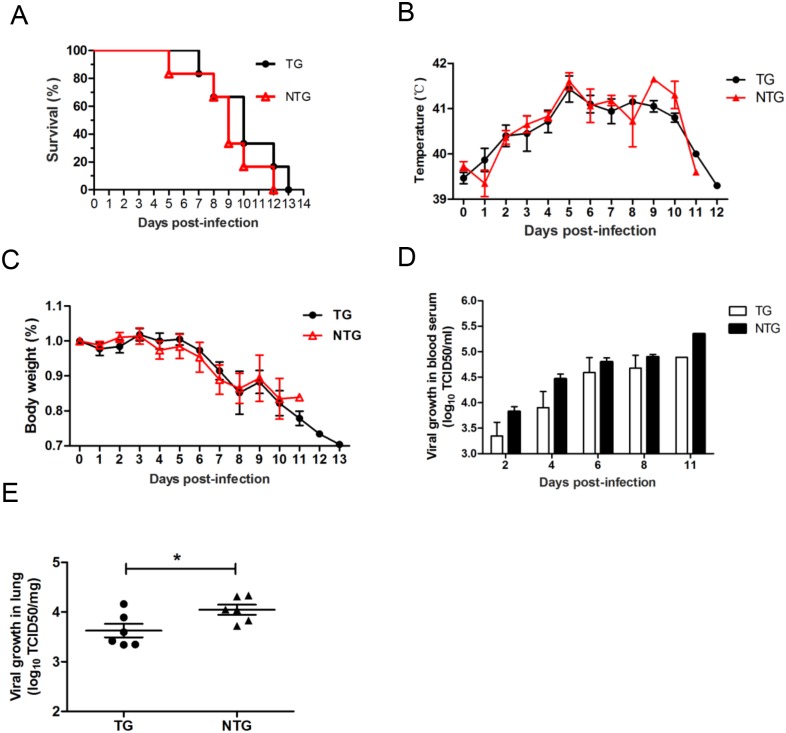
Transgenic pigs developed a lower level of viremia and had a lower virus load in the lungs. (A) Survival curves for the pigs in the two challenged groups after infection with PRRSV JXA1. The pigs in challenged TG group survived for a longer period than the pigs in the challenged NTG group (log rank test; P = 0.328). Group: challenged TG group, n = 6; challenged NTG group, n = 6. (B) Rectal temperature curves for the pigs from the two challenged groups after infection with PRRSV JXA1. (C) Body weight curves for pigs in the two challenged groups after infection with PRRSV JXA1. (D) Analysis of the viral loads in serum samples obtained from challenged TG and challenged NTG pigs at the indicated times. (E) Viral load in the lungs. The samples were collected from the lungs of dying challenged TG and challenged NTG pigs. The data in panels B, C, D and E are presented as the mean±SD. The data in panels B, C, D and E were analyzed using Student’s t-test. *, P<0.05.

The pigs that were directly infected with HP-PRRSV showed 100% lethality. The survival curves showed that the TG pigs survived longer (13 days) than the NTG pigs (12 days). Over the 13-day experimental period, the NTG pigs generally died 0–2 days earlier than the TG pigs (Fig 5A). All challenged pigs exhibited a high fever (over 40°C) at 2 days post-infection (dpi), and the body weight of the challenged pigs decreased at 4 dpi. The observed trends in body temperature and weight were similar in challenged TG and NTG pigs, and no significant differences were observed between TG and NTG pigs (Fig 5B and 5C). The viral loads in blood samples from the challenged TG group were lower than the loads in the challenged NTG group during the initial days of the experiment (Figs 4 and 5D). However, there was no significant difference between the two challenged groups. Notably, the average number of viral RNA copies in the lungs of challenged TG pigs was significantly lower than the average number in the lungs of challenged NTG pigs (Fig 5E).

**Fig 6:**
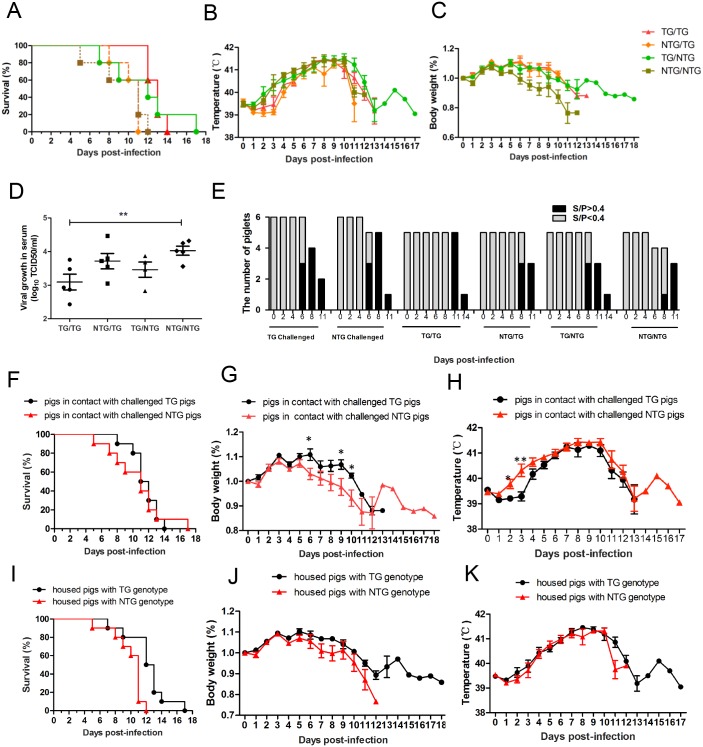
HDAC6 Overexpression enhanced resistance to infection during cohabitation with PRRSV-infected pigs. (A) The survival curves of the pigs housed with challenged pigs (log rank test; P = 0.153). n = 5 for each group. (B) Rectal temperature curves for the 4 groups housed with challenged pigs. (C) Body weight curves for the 4 groups housed with challenged pigs. The data were analyzed using ANOVA, which revealed that the body weights of the pigs in the NTG/NTG group were significantly lower than the body weights of the other groups after 5 dpi (P<0.05). (D) Analysis of the viral load in the serum samples obtained from pigs in the 4 housed groups at 4 dpi. (E) Antibody positive tests (S/P>0.4) indicating the level of the immune response *in vivo*. (F) Survival curves for the two groups of pigs that were housed in two separate rooms (log rank test; P = 0.712). (G) Body weight curves for pigs in the two groups that were housed in two separate rooms. The body weights of the pigs housed with NTG pigs were significantly higher than those of pigs housed with TG pigs at 6, 9 and 10 dpi. (H) Rectal temperature curves for the pigs from the two groups that were housed in two separate rooms. The rectal temperatures of the pigs housed with NTG pigs were significantly higher than were those of pigs housed with TG pigs at 2 and 3 dpi. (I) Survival curves for the housed pigs with different genotypes (log rank test; P = 0.046). (J) Rectal temperature curves for the housed pigs with different genotypes. (K) Body weight curves for the housed pigs with different genotypes. The data in panels B, C and D are presented as the mean±SD. The data in panels B, C and D were analyzed using ANOVA. The data in panels G, H, J, and K are presented as the mean±SE. The statistical significance of these data was analyzed using a t-test. *, P<0.05; **, P<0.01.

All pigs that were housed with PRRSV-injected pigs exhibited symptoms of HP-PRRSV infection, which is characterized by high fever, high morbidity, and high mortality [4]. Survival curve comparisons showed that pigs in the NTG/NTG group survived the shortest time among the in-contact pigs (Fig 6A). Furthermore, NTG/NTG pigs exhibited a significantly lower body weight than other in-contact pigs after 5 dpi (Fig 6C, P<0.05). The number of PRRSV RNA copies was determined in serum samples obtained at 2, 4, 6, 8, 11, 14 and 17 dpi. PRRSV RNA copies were detected in the serum samples of two of the ten pigs that were housed with challenged NTG pigs at 2 dpi. However, no PRRSV RNA copies were detected in the serum samples of pigs housed with challenged TG pigs (Fig 4). At 4 dpi, the average number of viral RNA copies was approximately 10-fold lower in the serum samples of pigs from the TG/TG group than in those of pigs from the NTG/NTG group (Fig 6D). In addition, ELISAs were used to determine the amount of PRRSV antibodies present in the serum. We found that pigs in TG/TG group did not show detectable PRRSV antibodies until 11 dpi and that PRRSV antibodies could be detected in other groups of housed pigs at 8 dpi (Fig 6E and S1 Table). In the case of natural infection, the clinical symptoms in the NTG/NTG group were significantly worse than those in the other housed groups.

Comparison of the survival ratio and the body temperatures for the in-contact pigs between with the TG and NTG challenged pigs (TG/TG and NTG/TG groups versus TG/NTG and NTG/NTG groups), in-contact pigs housed with NTG pigs showed the clinical symptoms of PRRSV infection prior to in-contact pigs housed with TG pigs (Fig 6F and 6H). Consistent with these results, the pigs in contact with challenged NTG pigs had remarkable lower body weights than the pigs housed in contact with challenged TG pigs at 6, 9, and 10 dpi (Fig 6G). These results indicated that the TG pigs exhibited altered infection transmission dynamics of JXA1 PRRSV virus transmission.

In addition, survival curves showed a significant difference between the in-contact pigs with different genotypes. The in-contact pigs with a TG genotype (TG/TG and TG/NTG) lived longer (17 dpi) than NTG in-contact pigs (NTG/TG and NTG/NTG) (12 dpi) (Fig 6I, log-rank test; P = 0.0046). The in-contact pigs with a TG genotype exhibited higher body weights and lower temperatures than those in-contact pigs with a NTG genotype after 6 dpi; however, this difference was not significant (Fig 6J and 6K). These results confirm that *HDAC6* overexpression in TG pigs improved resistance to PRRSV.

### *HDAC6* Overexpression suppresses the elevation of Actub in PRRSV-infected cells

**Fig 7:**
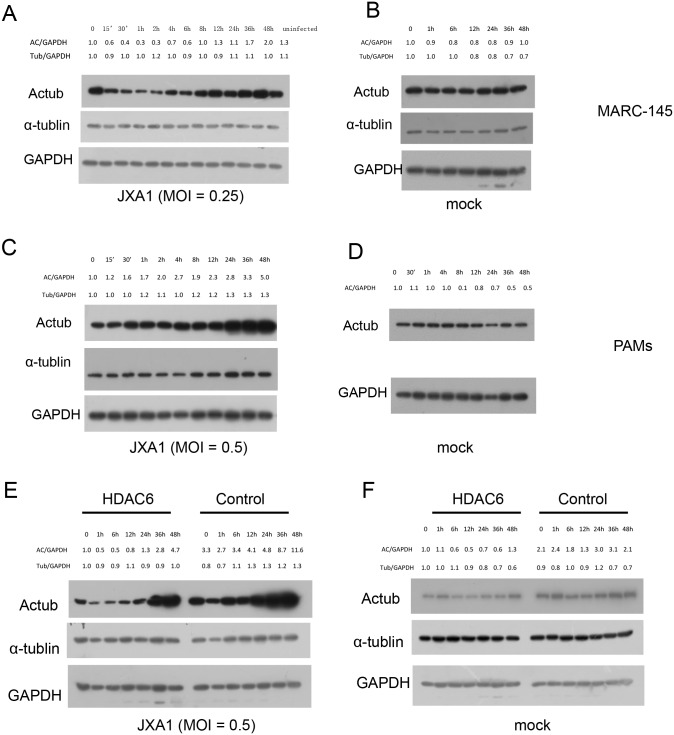
The effect of PRRSV and *HDAC6* overexpression on α-tubulin acetylation. Kinetic analysis of JXA1-induced α-tubulin acetylation in MARC-145 cells (A) and in untreated cells (B). Kinetic analysis of JXA1-induced α-tubulin acetylation in PAMs (C) and in untreated cells (D). Kinetic analysis of JXA1-induced α-tubulin acetylation in HDAC6-transfected MARC-145 cells (E) and in untreated cells (F). The α-tubulin acetylation or α-tubulin content was quantified and is presented as a ratio relative to the total amount of GAPDH (Actub/GAPDH or tub/GAPDH).

To further examine the potential role of HDAC6 in the PRRSV life cycle, we examined the capacity of PRRSV to induce the acetylation of α-tubulin, which is the substrate of HDAC6. We first examined the effects of PRRSV infection on α-tubulin acetylation in infected cells. The results showed that HP-PRRSV (JXA1) triggered the acetylation of α-tubulin in both MARC-145 cells (Fig 7A) and PAMs (Fig 7C). Maximal 2- and 5-fold increases were detected 48 h after exposure to JXA1. Similarly, elevated levels of Actub were also detected in MARC-145 cells and PAMs that were infected with the CH-1a strain (S4A and S4B Fig). No increase in the level of Actub was observed in uninfected cells (Fig 7B and 7D), suggesting that an increase in Actub levels is required for PRRSV infection. We then examined Actub levels in MARC-145 cells that were transfected with plasmids overexpressing *HDAC6* or a mock control. The elevation in Actub levels was inhibited in MARC-145 cells that overexpressed *HDAC6* (Fig 7E and 7F). These results imply that HDAC6 might exert its anti-viral activity by suppressing the level of Actub in PRRSV-infected cells.

## Discussion

As reviewed by [33], transgenic technology will allow for the generation of animals that are more resistant to infectious diseases. In recent years, many transgenic animals that can suppress viral infection and transmission have been developed [34,35]. Furthermore, a recent report has declared that replication of PRRSV is inhibited in TG pigs that constitutively express PRRSV-specific short interference RNA (siRNA) [25]. A novel alternative strategy is required to develop pigs that are genetically resistant to PRRSV infection. Previous studies have demonstrated the involvement of HDAC6 in viral life cycles. However, no reports have been published regarding the relationship between PRRSV and HDCA6. In the present study, we demonstrate that *HDAC6* overexpression leads to genetic resistance to PRRSV both *in vitro* and *in vivo*.

Although a number of cell lines overexpressing *HDAC6* have been established [11,12,36], animal models of *HDAC6* overexpression are rare, particularly in pigs. In the present study, we successfully constructed a porcine *HDAC6* overexpression model with stable *HDAC6* expression over two generations. We verified the integration and expression of the introduced *HDAC6* using PCR and qRT-PCR, respectively (Fig 1B and 1C). Unfortunately, no commercial antibody against pig HDAC6 is currently available. We detected pig HDAC6 using two types of antibodies specific for the homologous human HDAC6 protein [37,19]; however, these antibodies could not detect the pig HDAC6 protein (data not shown). Therefore, we detected the expression of a GFP tag to verify the overexpression of HDAC6 protein (Figs 1D, 2D and S1 Fig). In addition, the level of Actub protein, which is a substrate of HDAC6 [11], was detected in cells and tissues from TG pigs. As expected, Actub levels in the cells and tissues of TG pigs were lower than that observed in NTG or WT pigs (S2A and S2B Fig). These results suggested enhanced HDAC6 protein expression in the TG pigs. Although the CMV promoter can drive transgene expression in a variety of organs and tissues, qRT-PCR and western blot results showed that HDAC6 expression was lower in lungs than that in heart and skin tissues (Fig 1C and 1D).

PAMs are the primary target cells of PRRSV invasion [38]. Therefore, we detected the number of viral RNA copies in PAMs. qRT-PCR analysis of PRRSV-infected PAMs confirmed that integrated *HDAC6* expression inhibited viral replication *in vitro* (Fig 3C, 3D and 3E). This inhibitory effect is consistent with reduced viral load in blood of *HDAC6* TG pigs *in vivo* (Fig 4). Similar results have been reported in previous studies. HDAC6 overexpression inhibited the acetylation of α-tubulin, and prevented HIV-1 envelope-dependent cell fusion and infection. Compared with WT type controls, the HIV DNA copy number was reduced 4-fold in HDAC6-overexpressing cells [19]. Moreover, when infected with IAV, mock control cells released approximately 3-fold more viral progeny in the culture medium than HDAC6-overexpressing cells [22].

The animal experiments in the present study demonstrated that *HDCA6* overexpression partially protects pigs from PRRSV infection (Figs 5 and 6). We infected the pigs with PRRSV using two *in vivo* methods. In the challenge study, the effect of direct infection with HP-PRRSV via intramuscular injection was compared between challenged TG and NTG groups. Although no significant differences in body weights and rectal temperatures were observed between the challenged TG and NTG pigs (Fig 5B and 5C), the TG pigs showed significantly lower levels of viral RNA in the lungs than in NTG pigs (Fig 5E). An in-contact study was conducted to investigate whether *HDAC6* overexpression minimizes susceptibility to PRRSV through contact with infected pigs, mimicking the natural path of PRRSV infection. Rectal temperature and the viral load in the serum were lower in the NTG/NTG group than those in the other three in-contact groups (Fig 6B and 6D), but the opposite trend was observed for body weight (Fig 6C).

These results indicated that the transmission of and susceptibility to PRRSV were partially repressed in TG pigs. Animal experiments to determine the effect of HDAC6 overexpression on viral life cycle have been reported only in TG mice. After TG mice overexpressing HDAC6 and aged-matched WT mice were infected with avian H5N1 influenza virus, an increased survival rate and notably reduced virus titers in the trachea were observed in TG mice. However, the body weight did not differ significantly between the surviving TG mice and WT mice [24].

The processes of virus entry into host cells rely on host cell membrane dynamics, which is controlled by the cytoskeleton [39]. In addition, many viruses induce microtubule acetylation to promote intracellular viral transport [40]. HDAC6 is an important regulator of microtubules [11]. Thus, it was proposed that HDCA6 is a potential regulator of viral life cycles. With respect to HIV, the overexpression of HDAC6 reduces viral infection and decreases virus–cell membrane fusion by reducing Actub levels [41,19]. In IAV-infected cells, the virus induces microtubule acetylation to facilitate its intracellular trafficking [37]. HDAC6 exerts its anti-IAV function by negatively regulating the trafficking of viral components via Actub to the host cell plasma membrane [22]. Other studies have shown that herpes simplex virus type 1 (HSV-1), human herpes virus 8 (HHV-8) and adenoviruses enhance microtubule acetylation to facilitate viral proliferation [42–44]. Therefore, we considered that elevated Actub levels might be beneficial for viral proliferation. Whether this phenomenon is common among virus-infected cells would be worth investigating in future studies. In the present study, an increase in the level of Actub was also observed in MARC-145 cells and PAMs that were infected with the two PRRSV strains (Fig 7A and 7C and S4 Fig). Furthermore, we found that the overexpression of *HDAC6* suppressed the elevation of Actub in PRRSV-infected cells (Fig 7E and 7F). These results implied that overexpression of HDAC6 resulted in anti-PRRSV activity via the regulation of Actub levels. Although the results obtained in the present study suggest that overexpression of *HDAC6* contributed to increased resistance to PRRSV infection in TG pigs and PAMs, the mechanism for this effect remains unclear. It will be interesting to study the molecular mechanism that underlies this inhibition and to determine what parts of the pathway are involved.

Moreover, a mild decline in Actub levels was initially observed in MARC-145 cells that were infected with PRRSV, and the level of Actub subsequently increased. However, this variation was not observed in PAMs (Fig 7 and S4 Fig). The observed differences in these results might be caused by different receptors for virus recognition on the surface of the two cell types [45–47]. There is an important relationship between the cytoskeleton and viral life cycles, but few studies about the relationship between PRRSV and the cytoskeleton have been conducted. These results provide evidence for future research regarding the interaction between PRRSV and the cytoskeleton.

Notably, *HDAC6* overexpression has potential risks for tumorigenesis. As reviewed by Aldana-Masangkay and Sakamoto [48], *HDAC6* expression was upregulated in diverse tumors and cancer cell lines. Additionally, *HDAC6*-null mice were more resistant to chemical carcinogen-induced skin tumor formation [49]. In the present study, the genetically modified pigs that overexpressed *HDAC6* were consistently healthy, and there was no statistically significant difference in average body weight between TG and NTG siblings (S3B Fig). Moreover, tumorigenesis was not observed in the TG pigs, which is consistent with findings in TG mice overexpressing *HDAC6* [24]. However, additional studies are required to determine the long-term effects of *HDAC6* overexpression.

The results of the present study suggest that overexpression of *HDAC6* enhances resistance to PRRSV *in vivo* and *in vitro*. Thus, the present study provides evidence that supports the further study of interactions between PRRSV and HDAC6, which will facilitate the development of novel therapies against PRRSV.

## Supporting Information

S1 Fig The expression of GFP protein was detected in F1 transgenic pigs. The samples were collected from lung biopsies of three TG (pig nos. 1149, 1180, 1263) and two sibling NTG (pig nos. 1148, 1167) pigs. The protein samples were probed with an anti-GFP antibody. GAPDH was used as an internal control for western blot analysis.
(TIF)

S2 Fig The levels of acetylated α-tubulin were elevated in transgenic pigs. (A) Western blot analysis of F0 transgenic pigs. The samples were collected from heart and lung biopsies obtained from TG (No. 1) and wild-type (WT) pigs. The protein samples were probed with an anti-Actub antibody. (B) Western blot analysis of F1 pigs. The samples were collected from lung biopsies of four TG and three sibling NTG pigs. The protein samples were probed with an anti-Actub antibody. GAPDH was used as an internal control for qRT-PCR and western blot analysis.
(TIF)

S3 Fig Rectal temperature and body weight were normal in unexposed controls. (A) Rectal temperature curves and (B) body weight curves for the unexposed control pigs (n = 5 for each group). There were no significant differences in the rectal temperature and body weight between the TG and NTG pigs, which as the unexposed controls.
(TIF)

S4 Fig The levels of Actub were elevated in CH-1a-infected cells. Kinetic analysis of CH-1a-induced α-tubulin acetylation in MARC-145 cells (A) and in PAMs (B). The α-tubulin acetylation or α-tubulin content was quantified. The content is presented as a ratio relative to the total amount of GAPDH (Actub/GAPDH or tub/GAPDH).
(TIF)

S1 Table Sex and age of pigs in the present study. ♂, male; ♀, female.
(DOCX)

S2 Table S/P values for the pigs in the present study. Antibodies specific to the PRRSV M protein were measured at the indicated time points using ELISA (HerdChek, Idexx Laboratories). S/P ratios<0.4 were considered negative.
(DOCX)
