# Supplementary figures and images for: Overexpression of Histone Deacetylase 6 Enhances Resistance to Porcine Reproductive and Respiratory Syndrome Virus in Pigs
Source: PLoS One. 2017 Jan 4;12(1):e0169317. doi: 10.1371/journal.pone.0169317 (PMC5215653; doi:10.1371/journal.pone.0169317)

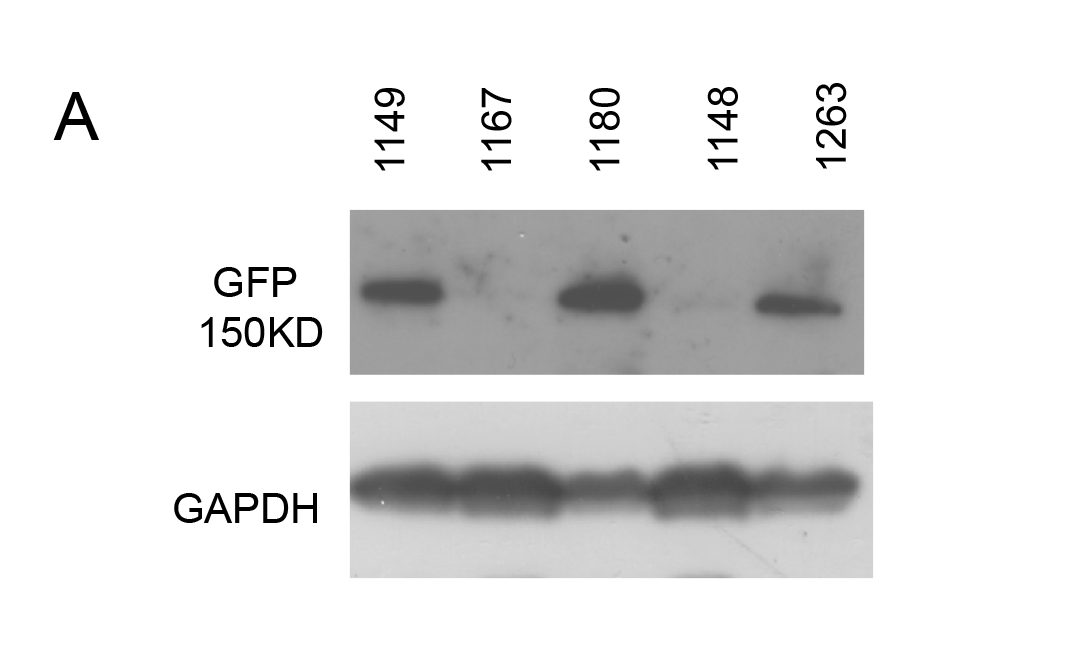

Supplement: S1 Fig — The samples were collected from lung biopsies of three TG (pig nos. 1149, 1180, 1263) and two sibling NTG (pig nos. 1148, 1167) pigs. The protein samples were probed with an anti-GFP antibody. GAPDH was used as an internal control for western blot analysis. (TIF) [file pone.0169317.s001.tif]

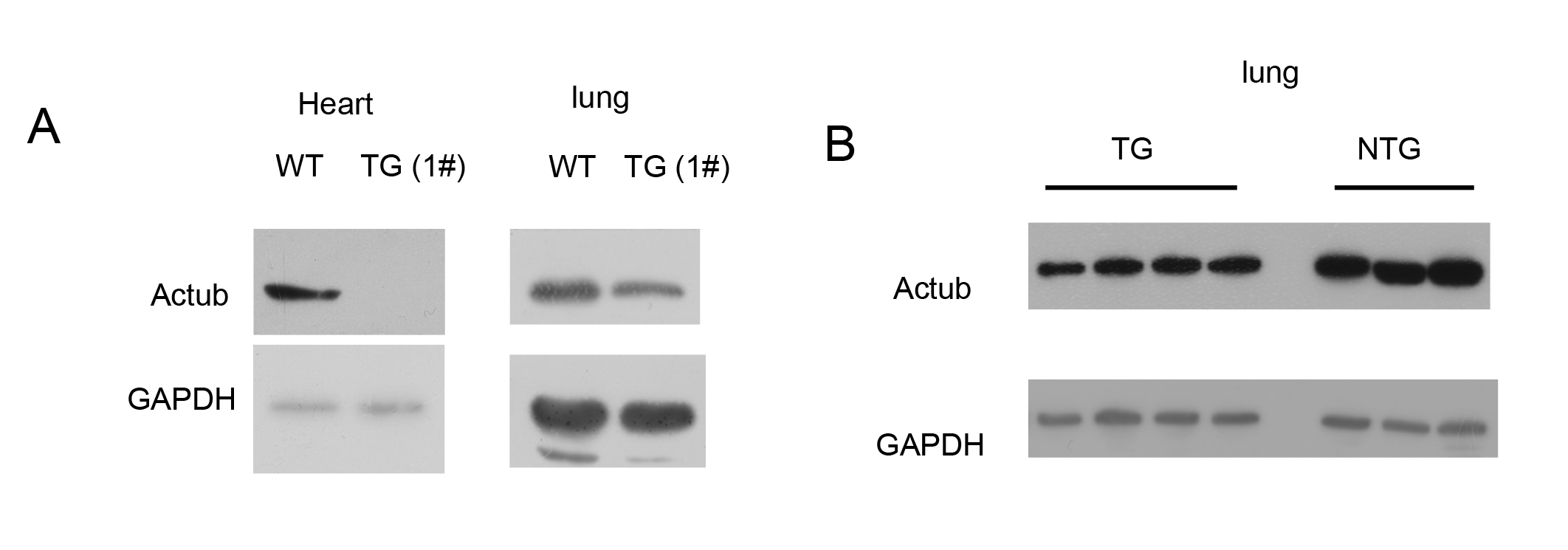

Supplement: S2 Fig — (A) Western blot analysis of F0 transgenic pigs. The samples were collected from heart and lung biopsies obtained from TG (No. 1) and wild-type (WT) pigs. The protein samples were probed with an anti-Actub antibody. (B) Western blot analysis of F1 pigs. The samples were collected from lung biopsies of four TG and three sibling NTG pigs. The protein samples were probed with an anti-Actub antibody. GAPDH was used as an internal control for qRT-PCR and western blot analysis. (TIF) [file pone.0169317.s002.tif]

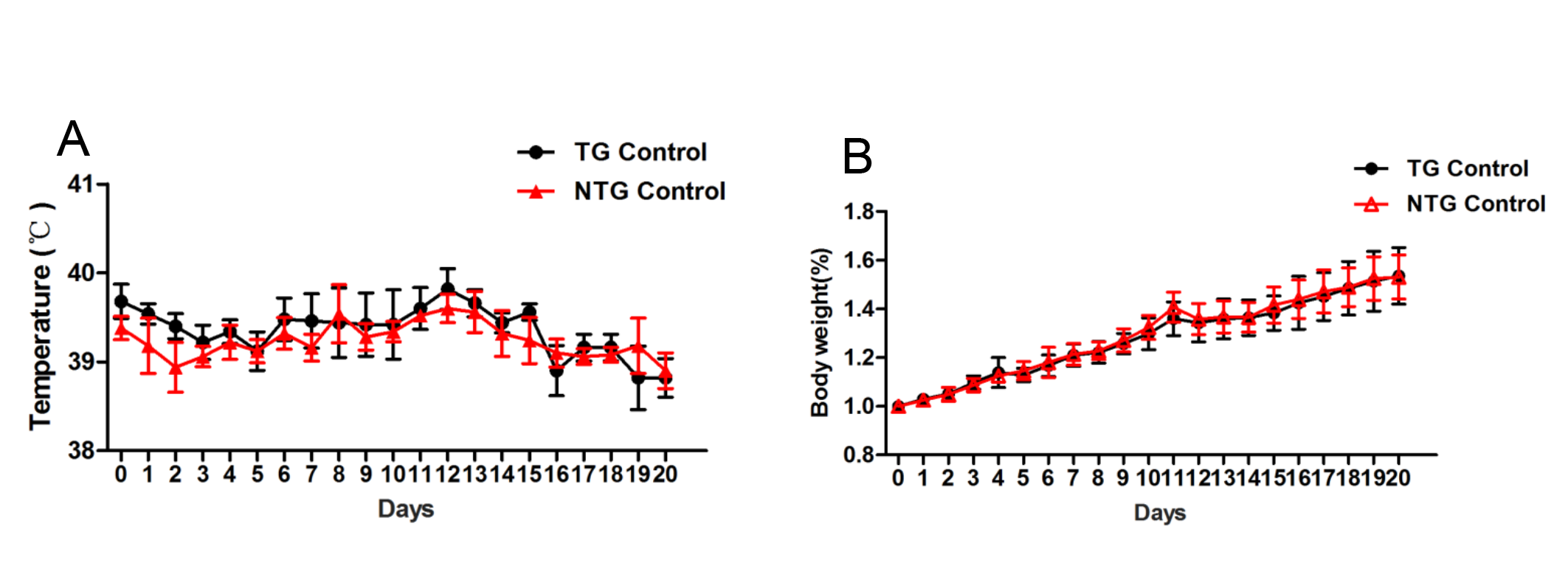

Supplement: S3 Fig — (A) Rectal temperature curves and (B) body weight curves for the unexposed control pigs (n = 5 for each group). There were no significant differences in the rectal temperature and body weight between the TG and NTG pigs, which as the unexposed controls. (TIF) [file pone.0169317.s003.tif]

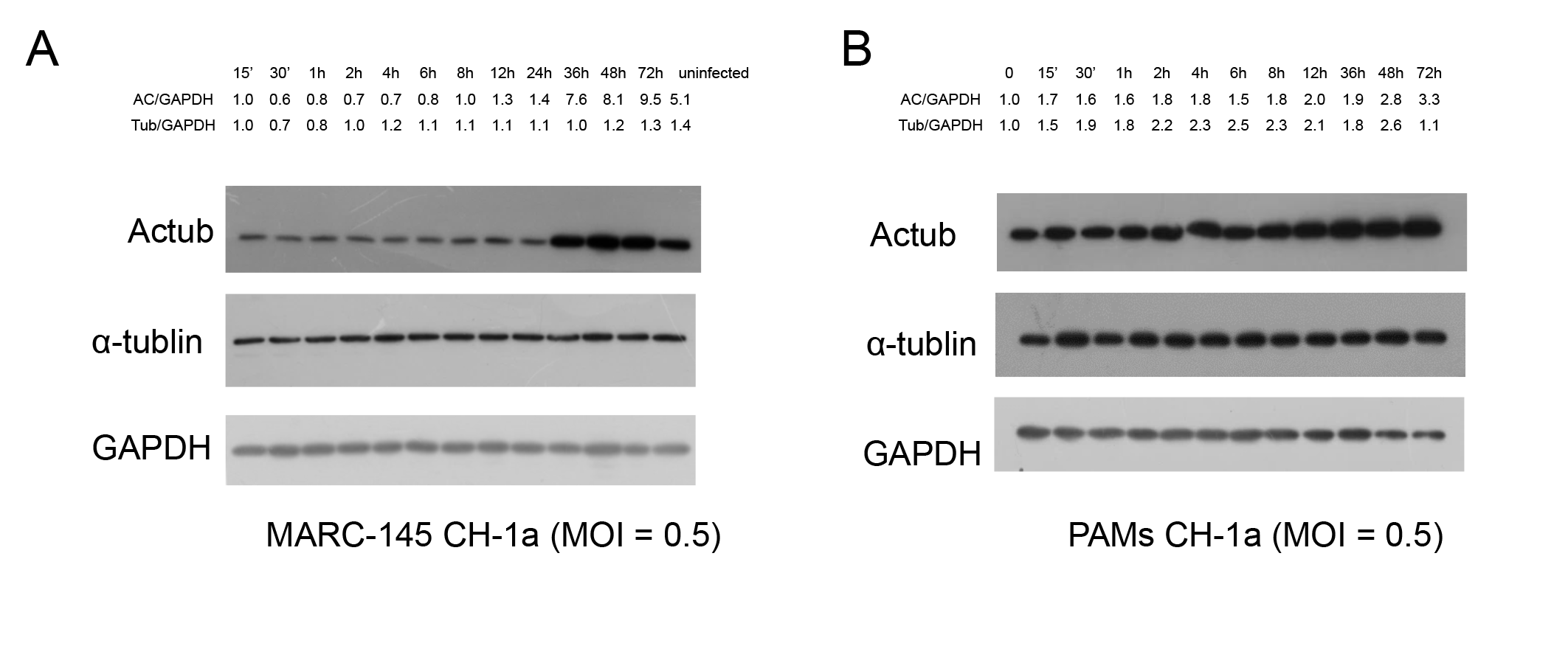

Supplement: S4 Fig — Kinetic analysis of CH-1a-induced α-tubulin acetylation in MARC-145 cells (A) and in PAMs (B). The α-tubulin acetylation or α-tubulin content was quantified. The content is presented as a ratio relative to the total amount of GAPDH (Actub/GAPDH or tub/GAPDH). (TIF) [file pone.0169317.s004.tif]

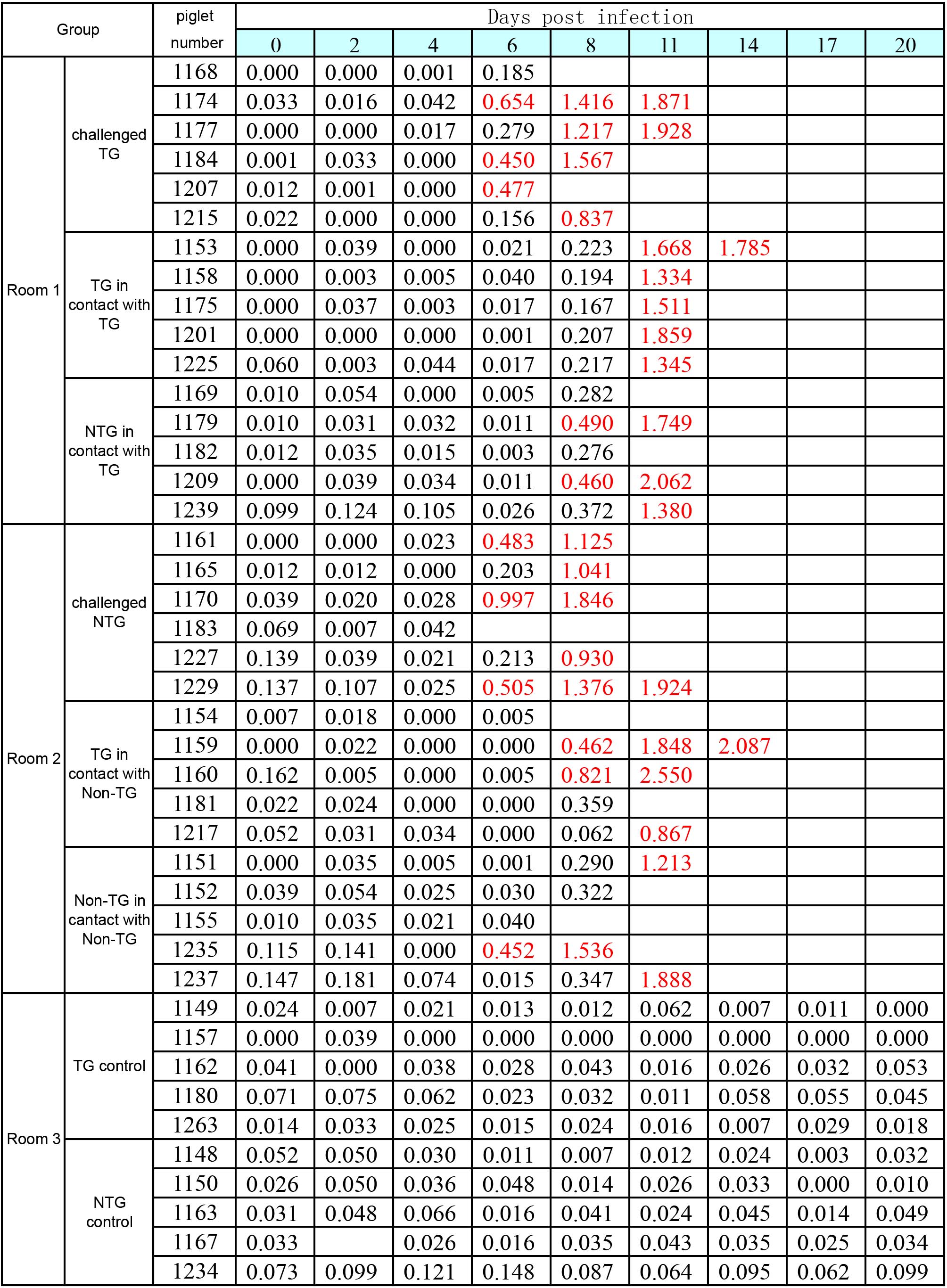


**Table S2. S/P values for the pigs in the president study.**

Supplement: S2 Table — Antibodies specific to the PRRSV M protein were measured at the indicated time points using ELISA (HerdChek, Idexx Laboratories). S/P ratios<0.4 were considered negative. (DOCX) [file pone.0169317.s006.docx]
