# Supplementary material for: Overexpression of Histone Deacetylase 6 Enhances Resistance to Porcine Reproductive and Respiratory Syndrome Virus in Pigs
Source: PLoS One. 2017 Jan 4;12(1):e0169317. doi: 10.1371/journal.pone.0169317 (PMC5215653; doi:10.1371/journal.pone.0169317)
Supplement: S1 Table — ♂, male; ♀, female. (DOCX) [file pone.0169317.s005.docx]

| Group | | piglet number | sex | age (day) |
| --- | --- | --- | --- | --- |
| Room 1 | challenged TG | 1168 | ♂ | 46 |
|  |  | 1174 | ♀ | 46 |
|  |  | 1177 | ♀ | 46 |
|  |  | 1184 | ♂ | 46 |
|  |  | 1207 | ♂ | 50 |
|  |  | 1215 | ♀ | 53 |
|  | TG in contact with TG | 1153 | ♀ | 46 |
|  |  | 1158 | ♀ | 46 |
|  |  | 1175 | ♂ | 46 |
|  |  | 1201 | ♀ | 50 |
|  |  | 1225 | ♂ | 50 |
|  | NTG in contact with TG | 1169 | ♂ | 46 |
|  |  | 1179 | ♀ | 46 |
|  |  | 1182 | ♀ | 46 |
|  |  | 1209 | ♂ | 50 |
|  |  | 1239 | ♂ | 53 |
| Room 2 | challenged NTG | 1161 | ♂ | 46 |
|  |  | 1165 | ♀ | 46 |
|  |  | 1170 | ♂ | 46 |
|  |  | 1183 | ♂ | 46 |
|  |  | 1227 | ♀ | 53 |
|  |  | 1229 | ♀ | 53 |
|  | TG in contact with Non-TG | 1154 | ♂ | 46 |
|  |  | 1159 | ♀ | 46 |
|  |  | 1160 | ♂ | 46 |
|  |  | 1181 | ♂ | 46 |
|  |  | 1217 | ♀ | 53 |
|  | Non-TG in cantact with Non-TG | 1151 | ♀ | 46 |
|  |  | 1152 | ♂ | 46 |
|  |  | 1155 | ♀ | 46 |
|  |  | 1235 | ♂ | 46 |
|  |  | 1237 | ♂ | 53 |
| Room 3 | TG control | 1149 | ♀ | 46 |
|  |  | 1157 | ♀ | 46 |
|  |  | 1162 | ♀ | 46 |
|  |  | 1180 | ♀ | 46 |
|  |  | 1263 | ♂ | 53 |
|  | NTG control | 1148 | ♂ | 46 |
|  |  | 1150 | ♀ | 46 |
|  |  | 1163 | ♀ | 46 |
|  |  | 1167 | ♀ | 46 |
|  |  | 1234 | ♂ | 53 |

**S1 Table. Sex and age of pigs in the present study.**
